# Prostatic arterial embolization for the treatment of lower urinary tract symptoms due to large (>80 mL) benign prostatic hyperplasia: results of midterm follow-up from Chinese population

**DOI:** 10.1186/s12894-015-0026-5

**Published:** 2015-04-16

**Authors:** Mao Qiang Wang, Li Ping Guo, Guo Dong Zhang, Kai Yuan, Kai Li, Feng Duan, Jie Yu Yan, Yan Wang, Hai Yan Kang, Zhi Jun Wang

**Affiliations:** Department of Interventional Radiology, Chinese PLA General Hospital Beijing, 100853 Beijing, People’s Republic of China

**Keywords:** Angiography, Benign prostatic hyperplasia (BPH), Lower urinary tract symptoms (LUTS), Prostatic artery embolization (PAE)

## Abstract

**Background:**

Currently, large prostate size (>80 mL) of benign prostatic hyperplasia (BPH) still pose technical challenges for surgical treatment. This prospective study was designed to explore the safety and efficacy of prostatic arterial embolization (PAE) as an alternative treatment for patients with lower urinary tract symptoms (LUTS) due to largeBPH.

**Methods:**

A total of 117 patients with prostates >80 mL were included in the study; all were failure of medical treatment and unsuited for surgery. PAE was performed using combination of 50-μm and 100-μm particles in size, under local anaesthesia by a unilateral femoral approach. Clinical follow-up was performed using the international prostate symptoms score (IPSS), quality of life (QoL), peak urinary flow (Qmax), post-void residual volume (PVR), international index of erectile function short form (IIEF-5), prostatic specific antigen (PSA) and prostatic volume (PV) measured by magnetic resonance (MR) imaging, at 1, 3, 6 and every 6 months thereafter.

**Results:**

The prostatic artery origins in this study population were different from previously published results. PAE was technically successful in 109 of 117 patients (93.2%). Follow-up data were available for the 105 patients with a mean follow-up of 24 months. The clinical improvements in IPSS, QoL, Qmax, PVR, and PV at 1, 3, 6, 12, and 24 months was 94.3%, 94.3%, 93.3%, 92.6%, and 91.7%, respectively. The mean IPSS (pre-PAE vs post-PAE 26.0 vs 9.0; *P* < .0.01), the mean QoL (5.0 vs 3.0; *P* < 0.01), the mean Qmax (8.5 vs 14.5; *P* < 0.01), the mean PVR (125.0 vs 40.0; *P* < 0.01), and PV (118.0 vs 69.0, with a mean reduction of 41.5%; *P* < 0.01 ) at 24-month after PAE were significantly different with respect to baseline. The mean IIEF-5 was not statistically different from baseline. No major complications were noted.

**Conclusions:**

PAE is a safe and effective treatment method for patients with LUTS due to large volume BPH. PAE may play an important role in patients in whom medical therapy has failed, who are not candidates for open surgery or TURP or refuse any surgical treatment.

## Background

Lower urinary tract symptoms (LUTS) are common complaints resulting from benign prostatic hyperplasia (BPH), is one of the most common diseases of aging men [1,2]. LUTS can reduce quality of life by impeding normal activities and causing complications such as acute urinary retention or urinary tract infection. The indication for treatment depends on the severity and bother of urinary symptoms. Treatment options include medical treatment, minimally invasive management, and surgical therapies.

Although both medical and surgical therapies for syptomatic BPH are effective, they are associated with significant morbidity rates and some degree of sexual dysfunction [3,4]. In addition, patients with LUTS due to BPH are often elderly and some patients may have severe comorbidities. Because of the increasing operative risk of undergoing transurethral resection of the prostate (TURP) or open surgery for these patients, especially in patients with large-volume BPH (>80 mL) [5,6], non-surgical treatment alternatives are required to meet their needs. Several minimally invasive treatments were originally conceived as an attempt to offer equivalent efficacy as operative therapy but without the burden and risk of operative morbidity [7,8]. Therefore, the development of new minimally invasive modalities for treatment of BPH has constituted an interesting field of research.

Recently, prostatic artery embolisation (PAE) for BPH has been shown to be a safe and effective procedure that improves lower urinary tract symptoms related to BPH and is associated with a decrease in prostate volume [9-11]. However, the rate of clinical failure after PAE was relatively high. As many as 25% of patients may not show a significant reduction in the international prostate symptoms score (IPSS) or improvement in peak flow rate (Qmax). In addition, the average of reduction rate in the prostatic volume after PAE varies from 20% to 32% [9-12]. One component of PAE where best practice remains to be defined is the choice of embolic agent size. In theory, embolization with larger particles (ie, >200 μm), as previously reported results [10,11], may not a optimal size for PAE because of early proximal occlusion. We assumed that smaller-size particles (<100 μm) may induce greater ischemia with a more distal penetration into the prostate, and hence lead to a better clinical outcome. In the present study, we designed to investigate the safety and efficacy of PAE with combined polyvinyl alcohol particles (PVA) 50-μm and 100-μm in size as a primary treatment for patients with LUTS due to large-volume BPH after failure of medical treatment.

## Methods

### Study population

#### Ethics statement

This prospective study was approved by the hospital review boards of Chinese Peoples Liberation Army General Hospital, and has been performed in accordance with the ethical standards laid down in the 1964 Declaration of Helsinki and its later amendments. Written informed consent was obtained from all the patients for the study.

From February 2009 to July 2013, a total of 117 patients (age range, 57–87 years; mean, 71.5 years) diagnosed with severe LUTS due to large-volume BPH (>80 mL) that was refractory to medical treatment underwent PAE. The base line data of these patients were provided in Table 1.

**Table 1:** **Pre-PAE baseline data (N = 117)**

**Characteristics**	**Values Mean** **± SD**	**Range**
Age (year)	71.5 ± 13.5	57.0–87.0
IPSS (point)	26.0 ± 5.5	21.0-35.0
QoL score	5.0 ± 1.0	4.0-6.0
PV (mL)	118.0 ± 35.0	86.0-164.0
PSA (ng/mL)	3.9 ± 3.0	1.0-7.2
Qmax (mL/s)	8.5 ± 2.0	5.0-10.0
PVR (mL)	125.0 ± 50.0	85.0-180.0
IIEF-5 (point)	11.0 ± 6.5	5.0-17.0

International Index of Erectile Function short form = IIEF-5, IPSS = International Prostate Symptom Score, PAE = prostaic arterial embolization, PSA = prostatic specific antigen, PV = prostatic volume, PVR = postvoid residual urine, Q_max=_peak urinary flow rate, QoL = quality of life.

Inclusion criteria included men older than 50 years with a diagnosis of severe LUTS (International Prostate Symptom Score [IPSS] >18 points, quality of life [QoL] score >3, Qmax <12 mL/sec) due to BPH refractory to medical treatment for at least 6 months (alpha-1-adrenergic receptor antagonist or/and 5-alpha-reductase inhibitor) and a prostatic volume (PV) >80 mL (86-164 mL). The patient selection was achieved in a multidisciplinary manner in conjunction with urologists and interventional radiologists. All patients were assessed by an urologist and anesthesiologist as being unsuited for surgery owing to pulmonary disease (chronic obstructive pulmonary disease [COPD] in 33 patients) and cardiovascular diseases on antiplatelet therapy (coronary artery stent placement in 57, coronary bypass in 14 and cardiac valve replacement in 3 patients). Fifteen patients underwent transrectal US-guided prostate biopsy due to a PSA level >4.0 ng/mL with negative results for malignancy. Exclusion criteria included malignancy, large bladder diverticula (>5 cm), large bladder stones (>2 cm), chronic renal failure, active urinary tract infection, neurogenic bladder and detrusor failure, urethral stricture, and unregulated coagulation parameters.

#### Patient evaluation

Efficacy variables of IPSS, QoL score (scored as delighted = 0, pleased = 1, mostly satisfied = 2, mixed-about equally satisfied and dissatisfied = 3, mostly dissatisfied = 4, unhappy = 5, and terrible =6), the International Index of Erectile Function short form (IIEF-5), Qmax, post-void residual volume (PVR), and PV were assessed before PAE and at 1, 3, 6 and every 6 months after the procedure. Serum prostatic specific antigen (PSA) was assessed before PAE and at 24 hours, 1 week, 1, 3, 6 and every 6 months after the procedure. The PV was measured by magnetic resonance (MR) imaging. The MR imaging protocol for all examinations was the same, including axial and sagittal T2-weighted and non–contrast-enhanced and contrast-enhanced T1-weighted pulse sequences, and a 1.5-T magnet was used with a phased-array 12-channel body coil (GE Healthcare, Milwaukee, Wisconsin). The volume of prostate was determined using the standard ellipsoid formula: length × width × height × 0.52. All MR images were assessed independently by two radiologists who were unaware of the outcomes of PAE, and disparate measurements were resolved by consensus.

#### Embolization technique

Patients stopped taking all prostatic medications 3 days before embolization. After undergoing successful PAE, all prostatic medications were stopped during the entire follow-up period if there was consistent clinical improvement. Patients started an acid-suppressing drug (omeprazole 20 mg, AstraZeneca Pharmaceutical Co. Ltd., China, once daily), an anti-inflammatory (naproxen 750 mg, Guangzhou Baiyun Mountain Pharmaceutical Co. Ltd., China, twice daily) and an antibiotic (ciprofloxacin, 500 mg, Guangzhou Xin Pharmaceutical Co. Ltd., China, twice daily) 1 day before the procedure and continued for 7 days following PAE. During PAE, we did not us the analgesic drugs routinely because all the patients were well tolerated to the procedures.

#### Angiography

Patients underwent angiography and PAE in a therapeutic angiography unit equipped with a digital flat-panel detector system (INNOVA 4100 IQ; GE Healthcare, Milwaukee, Wis, USA) with nonionic contrast medium (Visipaque 320 mgI/mL; GE Healthcare). Embolization was performed with a unilateral femoral approach in all patients. After local anesthesia was achieved, the femoral artery was cannulated using a 4-Fr vascular sheath (Radifocus, Terumo, Japan) with Seldinger’s technique.

Initial pelvic angiography was performed with a 4-Fr pigtail type catheter (Cordis, USA) to evaluate iliac vessels. Selective digital subtraction angiography (DSA) was performed with a 4-Fr Simmons I catheter (Cordis, USA) to evaluate the hypogastric and prostatic arteries (PAs) by using the ipsilateral anterior oblique projection of 30^o^. The PAs were identified with DSA and Cone-beam computed tomography (CB-CT), and selectively catheterized with a coaxial 2.7-F microcatheter (Progreat 2.7; Terumo, Tokyo, Japan). Selective PA angiography before embolization was performed (3–5 mL contrast medium at 0.5-1 mL/s) in neutral and ipsilateral anterior oblique projections (35^o^) to ensure that the tip of the microcatheter was inside or at the ostium of the prostatic arteries. CB-CT was performed with a 3–5-second delay after injection of 4–6 mL contrast medium at 0.5-1 mL/s to evaluate for sites of nontarget embolization.

The origin of the prostatic arteries, revealed by the DSA, rotational angiography (images from a rotational scan acquired with a C-arm equipped with a flat panel detector) and Cone-beam CT, was assessed independently by two interventional radiologists with more than 10 years of experience; and the disparate findings were resolved by consensus.

#### Embolization

We started PAE with smaller PVA particles (47 ~ 90-μm, mean 50-μm; Polyvinyl alcohol foam embolization particles, PVA, Cook Incorporated, Bloomington, IN, USA)) for the distal or intra-prostate embolization; when reaching near stasis in the intra-prostate arterial branchese, we switched to larger PVA particles (90 ~ 180-μm, mean 100-μm; PVA, Cook Incorporated, Bloomington, IN, USA) for the proximal of the prostatic arterial embolization. This technique was modified from the suggestion by Bilhim T et al. [13]. We believe that using the smaller-sized particles firstly is essential to avoid early proximal occlusion of the prostatic arteries and to achieve the goal of diffuse gland parenchymal ischemia.

Each vial of PVA (1 mL) was diluted in a 40-mL solution of nonionic contrast medium (iodixanol 320 mgI/mL; Visipaque; GE Healthcare)*.* The particles were slowly injected through a 2-mL syringe under fluoroscopic control. Before embolization, vasodilator with nitroglycerin (200-300 μg) was used intra-arterially through the microcatheter to prevent vasospasm and to increase artery size to facilitate super-selective catheterization. The end point of embolization was near stasis; after it was achieved, a waiting time of 4-5 min followed for the particles to be redistributed in the feeding vessels; and then more embolic material was injected until complete stasis of the feeding artery was seen fluoroscopically. After PAE, angiography was performed using the power injector, with the 4-F catheter at the anterior branch of the internal iliac artery to check for any further blood supply to the prostate. Embolization was then performed on the contralateral side by using the same technique.

#### Post-procedural management

The patients stayed in the hospital for 1-6 days for observation. The patients were monitored for adverse effects. Appropriate hydration was administered 2 to 3 days after PAE. In all individuals, antibiotics were given to prevent infection as described before.

#### Outcome measures

Technical success was defined as unilateral or bilateral PAE, as successful embolization of all angiographically and/or CBCT-visible arterial supply to the prostate. Primary end points were the reduction of 7 points of the IPSS (or at least reduction of 25 % of the total score) and the increase of Qmax (>3 mL/sec) at 24-month after PAE. Secondary end points were the reduction of PV, PVR, and QoL at 24 months after PAE. Clinical failure after PAE was defined when one of the following criteria was met: IPSS ≥ 20, QoL ≥ 4, Qmax improvement <3 mL/s.

Postembolization symptoms and complications were registered and classified according to the quality improvement guidelines for percutaneous transcatheter embolization [14]. Complications were considered minor if they could be addressed by ambulatory medical treatment and major if they resulted in prolonged hospitalization, hospital readmission, or required surgery.

#### Statistical analysis

The study’s quantitative variables were expressed as mean values, standard deviation, and minimum and maximum values, whereas the qualitative variables were expressed as numbers and percentages. A Student *t* test for paired samples was used when appropriate. A *P* value of 0.05 or lower was considered to indicate statistical significance. Statistical analysis was performed using SPSS 16.0 software for Windows (Chicago, Illinois).

## Results

PAE was technically successful in 109 of 117 patients (93.2%). Technical failure was seen in 8 patients (6.8%): the embolization was impossible owing to severe tortuosity and atherosclerotic changes of the iliac arteries in 6 patients, none of the prostatic arteries were revealed in 2 patients. Bilateral PAE was performed in 101 (92.7%) patients**;** the remaining 8 (7.3%) patients underwent unilateral PAE due to severe atherosclerotic stenosis of an unilateral PA. Mean procedural time was 105 min (range 65–180 min) with a mean fluoroscopy time of 30.0 min (range 20–45 min).

**Figure 1:**
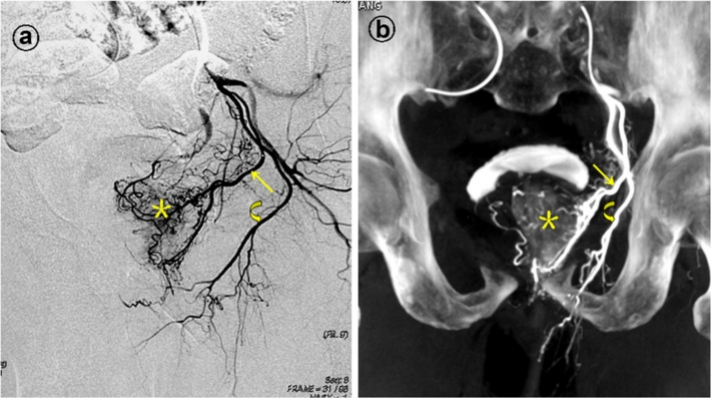
Prostatic artery arise from the gluteal-pudendal trunk. Images from a patient with significant lower urinary tract symptoms due to benign prostatic hyperplasia (92 mL) underwent bilateral PAE. **a**. Digital subtraction angiography (DSA) after selective catheterization of the anterior division of the left internal iliac artery with ipsilateral oblique view demonstrated the left prostatic artery (straight arrow) arising from gluteal-pudendal trunk; the curved arrow indicates the left internal pudendal artery; and the asterisk indicates the contrast staining in the left prostate lobe. **b**. Cone-beam CT image with coronal view after selective catheterization of the anterior division of the left internal iliac artery demonstrates the left prostatic artery (straight arrow) and the left internal pudendal artery (curved arrow). The asterisk indicates the contrast staining in the left prostate lobe.

**Figure 2:**
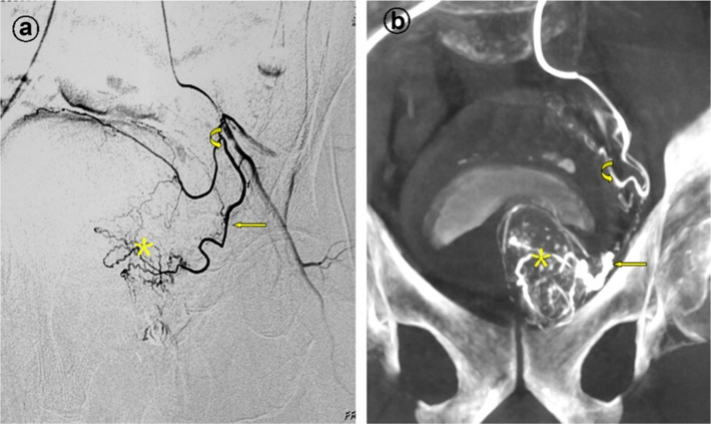
Prostatic artery arise from the superior vesical artery. Image from a patient with lower urinary tract symptoms due to benign prostatic hyperplasia (121 mL) underwent PAE. **a**. Digital subtraction angiography (DSA) of the anterior division of the left internal iliac artery with ipsilateral oblique view demonstrates the left prostatic artery (straight arrow) and the superior vesical artery (curved arrow). The asterisk indicates the corkscrew pattern of intra-prostate arteriola. **b**. Cone-beam CT image with coronal view after selective catheterization of the anterior division of the left internal iliac artery demonstrates the left prostatic artery (straight arrow) and the superior vesical artery (curved arrow). The asterisk indicates the corkscrew pattern of intra-prostate arteriola.

**Figures 3:**
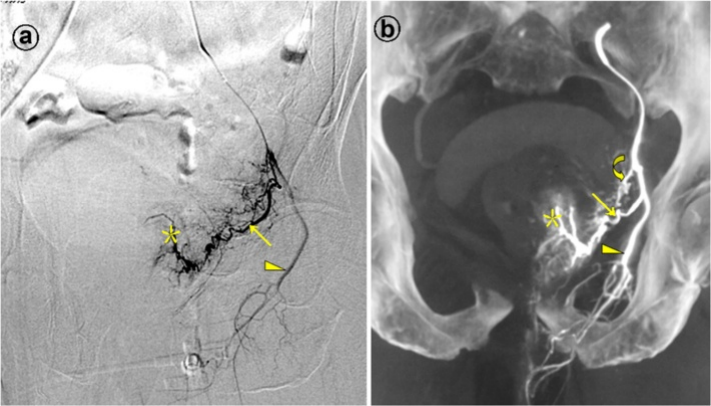
Prostatic artery arise from the internal pudendal artery. Images from a patient with severe lower urinary tract symptoms due to benign prostatic hyperplasia (117 mL) underwent PAE. **a**. Digital subtraction angiography (DSA) of the anterior division of the left internal iliac artery with ipsilateral oblique view demonstrates the left prostatic artery (straight arrow) and the left internal pudendal artery (arrowhead). The asterisk indicates the contrast staining in the left prostate lobe. **b**. Cone-beam CT image with coronal view after selective catheterization of the anterior division of the left internal iliac artery demonstrates the left prostatic artery (straight arrow) and the left internal pudendal artery (arrowhead). The curved arrow indicates the inferior vesical artery, which is difficult to identifying on the DSA. The asterisk indicates the contrast staining in the left prostate lobe.

Based on the analysis of selective DSA, rotational angiography, and CB-CT of the internal iliac arteries, it was possible to identify the number of independent PAs and their origin in 109 patients with 218 pelvic sides. There was one PA in 95.0% of the pelvic sides (207/218) and two independent PAs in 5.1% (11/218). The most frequent PA origin was the gluteal-pudendal trunk (39.5%; 86/218; Figure 1). Other common origins were the superior vesical artery (31.7%; 69/219; Figure 2), the middle third of internal pudendal artery (27.5%; 60/218; Figure 3). Three PAs (1.4%) arise from the middle rectal artery (Table 2).

**Table 2:** **Prostatic artery origin: 109 patients (218 pelvic sides)**

**PA orign**	**Incidence**
Gluteal-pudendal trunk	86 (39.5%)
Superior vesical artery	69 (31.7%)
Internal pudendal artery	60 (27.5%)
Middle rectal artery	3 (1.4%)

**Figures 4:**
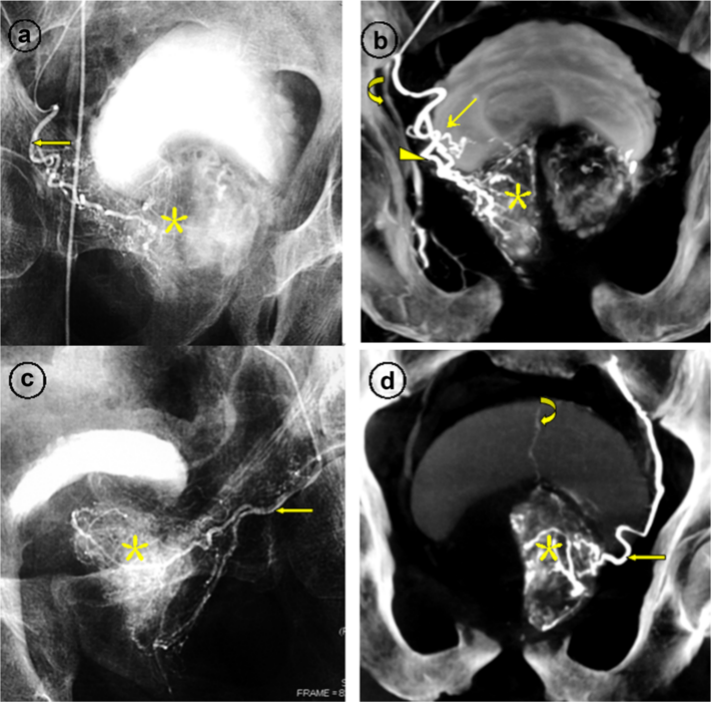
Images from a patient with lower urinary tract symptoms due to large benign prostatic hyperplasia (107 mL) underwent bilateral PAE. **a**. Angiography after selective catheterization of the riht prostatic artery (straight arrow) demonstrates contrast staining in the right prostate lobe (asterisk). **b**. Cone-beam CT image with coronal view after super-selective catheterization of the right prostatic artery demonstrates the the anterior-lateral prostatic branch (arrowhead), supplying to the central gland; the posterior-lateral prostatic branch (straight arrow), supplying to the peripheral and caudal gland. The asterisk indicates the contrast staining in the right prostate lobe and the curved arrow indicates the right internal pudendal artery. **c**. Angiography after super-selective catheterization of the left prostatic artery (straight arrow) demonstrates the corkscrew pattern of intra-prostate arteriola and contrast medium staining in the left prostate lobe (asterisk). **d**. Cone-beam CT image with coronal view after super-selective catheterization of the left prostatic artery (straight arrow) demonstrates contrast medium staining in the left prostate lobe (asterisk). The curved arrow indicates a branch of superior vesical artery, usually presented with high pressure injection of contrast medium through the anastomoses.

**Figures 5:**
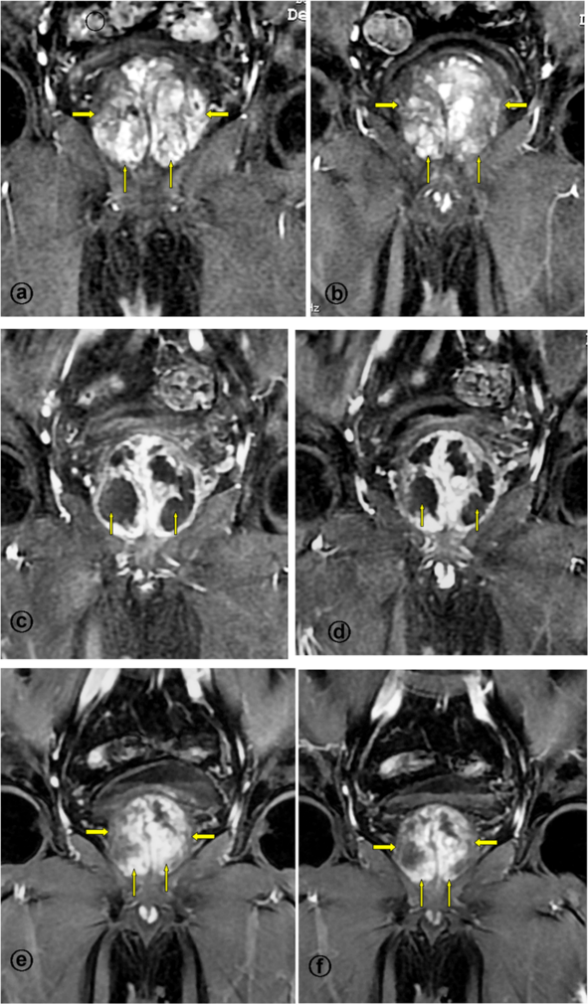
MR Images from a patient with lower urinary tract symptoms due to large benign prostatic hyperplasia underwent bilateral PAE, the same case as the Figure 4. **a**-**b**. Enhanced T1-weighted coronal MR images obtained before PAE shows a large benign prostatic hyperplasia (straight arrows). **c**-**d**. Enhanced T1-weighted coronal MR images obtained at 1-month after PAE shows significantly infarct areas on the both side of the prostate (straight arrows), with the volume reduction of 12%. **e**-**f**. Enhanced T1-weighted coronal MR images obtained at 12-month after PAE shows the prostate volume reduction of 62%; this patient experienced marked clinical improvement during 32 months follow-up, with IPSS improvement of 85%.

Follow-up data were available for the 105 patients, who were observed for a mean of 24 months (range 17–36 months). Four patients were lost to follow-up. The proportion of patients who demonstrated clinical success at 1, 3, 6, 12, and 24 months was 94.3% (99 of 105 patients), 94.3% (99/105 patients), 93.3% (98 of 105 patients), 92.6% (87 of 94 patients), and 91.7% (77 of 84 patients), respectively. As shown in Table 3, the LUTS of the patients showed significant improvements. Significant infarcts (mean 60%, range 55 %-90 %) were seen in all patients with clinical success as measured by MRI at 1-month after PAE, exclusively in the prostatic central zone; the infarct areas were reduced progressively in size. At 6-12 months after PAE, the infarcts could not be detected clearly in the majority of patients, resulting from the netrotic tissue absorption (Figures 4 and 5).

**Table 3:** **Clinical values over time of response variables after PAE**

**Variable**	**1 Mo (n = 105)**	**3 Mo (n = 105)**	**6 Mo (n = 105)**	**12 Mo (n = 94)**	**24 Mo (n = 84)**	
	**Mean** **± SD**	**Mean** **± SD**	**Mean** **± SD**	**Mean** **± SD**	**Mean** **± SD**	***P*** **Values**
Age(year)	71.5 ± 12.5	71.5 ± 12.5	71.5 ± 12.5	72.5 ± 11.5	70.5 ± 11.0	_
IPSS(point)	9.5 ± 5.5	8.5 ± 3.0	7.5 ± 4.0	8.0 ± 4.5	9.0 ± 5.5	<0.01
QoL score	2.5 ± 1.0	3.0 ± 0.5	3.0 ± 1.0	2.5 ± 1.5	3.0 ± 1.0	<0.01
PV (mL)	103.8 ± 30.0	72.5 ± 25.0	70.0 ± 15.0	68.5 ± 15.0	69.0 ± 18.0	<0.01
Qmax (mL/s)	14.0 ± 3.5	15.0 ± 4.5	15.5 ± 6.5	14.5 ± 5.0	14.5 ± 3.5	<0.01
PVR (mL)	45.0 ± 20.0	40.0 ± 25.0	35.0 ± 15.0	40.0 ± 20.0	40.0 ± 15.0	<0.01
IIEF-5 (point)	11.0 ± 5.0	10.0 ± 4.0	12.0 ± 3.0	13.0 ± 2.0	10.0 ± 2.5	0.6

IIEF-5 = International Index of Erectile Function short form, IPSS = International Prostate Symptom Score, PSA = prostatic specific antigen, PV = prostate volume, PVR = postvoid residual urine, Q_max=_peak urinary flow rate, QoL = quality of life.

At 24-month follow-up of these 84 patients, the mean IPSS decreased from 26.0 ± 5.5 points to 9.0 ± 5.5 points (*P* < 0.01), mean QoL decreased from 5.0 ± 1.0 points to 3.0 ± 1.0 points (*P* < 0.01), mean Qmax increased from 8.5 ± 2.0 to 14.5 ± 3.5 mL/s (*P* < 0.01), mean PVR decreased from 125.0 ± 50.0 mL to 40.0 ± 15.0 mL (*P* < 0.01), and mean PV decreased from 118.0 ± 35.0 mL to 69.0 ± 18.0 mL (with a mean reduction of 41.5%, P < 0.01). Sixty-two patients were followed more than 24 months and these changes were sustained throughout the observation period. No significant differences (*P* = 0.6) were observed in IIEF-5 scores during the follow-up period compared with preoperative data.

The serum total PSA values before and after PAE were provided in Table 4. At 24 h after embolization, the mean serum total PSA increased from 4.00 ± 2.50 ng/mL to 87.50 ± 45.00 ng/mL (with s mean of 21.9 times relative to the mean baseline values; *P* < 0.01). By 1 week after embolization, mean PSA dropped to 30.5 ± 20.0 ng/mL (mean, 7.6 times; *P* < 0.01). By 1 month after embolization, mean PSA dropped to the baseline values (*P* = 0.6); by 3-month and 6-month of follow-up, the mean PSA was statistically significantly lower than at baseline (*P* < 0.05), and was almost sustained over time.

**Table 4:** **Total serum PSA values before and after PAE (n = 84)**

	**Values (ng/mL, Mean** **± SD)**	**Range**	***P*** **Values**
Pre-PAE	4.0 ± 2.5	1.2-6.5	-
24 h	87.5 ± 45.0	30.0-145.0	<0.01
1 week	30.5 ± 20.0	9.5-57.0	<0.01
1-Month	4.2 ± 2.5	1.5-6.0	0.6
3-Month	3.7 ± 1.6	0.8-4.5	0.04
6-Month	3.1 ± 1.5	1.0-4.5	0.03
12-Month	3.9 ± 2.5	0.7-4.9	0.05
18-Month	4.1 ± 1.5	1.0-4.6	0.05
24-Month	3.7 ± 1.5	1.5-4.7	0.05

PAE = prostaic arterial embolization, PSA = prostatic specific antigen.

Poor outcome after PAE was observed in 7 (8.3%) patients at 24 months after PAE: unilateral PAE in 6 patients and bilateral PAE in one patient. The PAS values in the 7 patients were increased by 4.9-8.5 times (mean, 7.0 times) relative to their mean baseline values at 24 h after embolization. The prostate infarction rate detected by MRI at 1 month after PAE in the 7 patients was 10%-25%; the PV reduction rate at 3-month follow-up was 10%-17% (mean, 15%). The clinical failure had direct relationship with the PAS values at 24 h after PAE, the prostate infarction rate at 1 month after PAE, and the PV reduction rate at 3-month follow-up.

No major adverse events were noted in this series. As minor complications (Table 5), urethral burning occurred in 19 (17.4%) patients, transient hematuria occurred in 11 (10.9%) patients, transient hemospermia occurred in 9 (8.1%) patients, transient rectal bleeding occurred in 8 (7.34%) patients, and small inguinal hematoma at the punctured site occurred in 3 (2.8%) patients. These patients with small amount of rectal bleeding may be attributed to ischemic rectal complication, resulted as the rectal nontarget embolization. All these minor complications disappeared during the first 1 week. Thirty-one patients (28.4%) experienced acute urinary retention at 1-3 days after PAE; for relief, a temporary bladder catheter was placed at the time for 3-6 days and the patients were able to void spontaneously before discharge. There were no incidences of ejaculatory disorders post-procedure. No other minor complications were observed.

**Table 5:** **Minor complications in the first week after PAE (n = 109)**

**Adverse event**	**Number of patients (%)**
Urethral burning	19 (17.4%)
Hematuria	11 (10.9%)
Hematospermia	9 (8.1%)
Rectal bleeding	8 (7.3%)
AUR	31 (28.4%)
Inguinal hematoma	3 (2.8%)

PAE = prostate arterial embolization, AUR = acute urinary retention.

## Discussion

The surgical management of patients with prostate volumes >80 mL causing LUTS secondary to BPH presents a challenge [15]. TURP has been the ‘gold standard’ surgical procedure during the last 30 years, but its role in treating patients with prostate volumes >80 mL is limited, mainly because of intra-operative and postoperative morbidities (e.g., intraoperative and postoperative bleeding, postoperative hyponatremia, and urethral stricture) [16,17]. Despite the more recent development of new techniques such as endoscopic laser enucleation, plasma enucleation, and laparoscopic adenomectomy, in terms of efficacy, open prostatectomy (OP) is still considered the “gold standard” for the surgical treatment of BPH in patients with prostates > 80 mL [1,2]. However, OP is associated with a high morbidity rate, considerable blood loss, prolongedrecovery time, and heavy patient burden [2]. Serretta et al. [18] reported 8.2% blood transfusion in a large Italian series of open prostatectomy for large prostates. Gratzke et al. [19] performed open surgery on 902 BPH patients with an average prostate volume of 96.3 ± 37.4 mL and found that the total incidence of postoperative complications reached 17.3%. Thus, the new treatment options are necessary to meet this challenge. Recently, PAE is emerging and is a promising minimally invasive therapy that improves lower urinary tract symptoms related to BPH and is associated with a decrease in PV [9-11].

Our study demonstrates that PAE could be used safely and effectively as a alternative treatment for BPH in patients with large volume BPH. Consistent with the literatures [9-11,20], our experience showed that PAE is a safe procedure, even in patients who were unsuited for surgery, without significant increases in morbidity or mortality. In the studies by Carnevale FC et al. [10], Bagla S et al. [20], and Pisco JM et al. [21], the mean prostatic volume before PAE was 69.7 mL (range 43.5-92 mL), 64 mL, and 83.5 mL (range 24-269 mL), respectively. In our study the mean prostate volume before PAE (118 mL, range 86-164 mL) was larger than that of the previous studies.

In the present study, the PV decreased from baseline to 24-month of follow-up (118.0 mL vs 69.0 mL, with a mean reduction of 41.5%, *P* <0.01), and Q_max_ increased (8.5 mL/s vs 14.5 mL/s, mean increase of 70.59%, *P* <0.01). This decrease in PV and increase in Q_max_ was accompanied by a significant reduction in BPH symptom burden as measured by IPSS (mean score, 26.0 at baseline, 9.0 in follow-up; *P* <0.01) and a commensurate improvement in patient QoL (mean index, 5.0 at baseline, 3.0 in follow-up; *P* <0.01). Many patients with LUTS due to large volume BPH are elderly, fragile patients with various comorbidities and therefore unsuited for surgery because of the operative risks involved [5,6]. The potential for PAE as an alternative treatment in patients with prostates > 80 mL is significant because TURP and laparoscopic prostatectomy are typically not considered for this population [1,2].

Comprehension of the functional arterial anatomy is crucial for an effective and a safe embolization, allowing better results and avoiding complications from untargeted embolization to surrounding organs (bladder, rectum, and penis) [22]. In a recent in vivo study by Bilhim T et al. [23], the authors reported that the origin of the prostatic artery is highly variable. PAs usually arise from the internal pudendal artery (35%), from a common origin with the superior vesical artery (20%), from the common anterior gluteal-pudendal trunk (15%), from the obturator artery (10%), or from a common prostato-rectal trunk (10%). Other origins are from the inferior gluteal artery, superior gluteal artery, or from an accessory pudendal artery (10%). Carnevale FC et al. [10] reported that the most common artery supplying the prostate was the inferior vesical artery, but branches from other arteries were also found to feed the gland. In the present study, we used the conventional DSA, combined with rotational angiography and CB-CT, for identifying the prostatic arteries and its origin; it may be more accurate and more reliable than the conventional DSA alone for evaluation the pelvic vascular anatomy [21]. Our findings of the prostatic artery origins were somewhat different from previously published results [10,23]. In this study, we found that 95.0% of the internal iliac artery had only one prostatic artery, 5.1% (11/218) had two independent prostatic arteries, 39.5% originated from the gluteal-pudendal trunk,31.7% originated from the superior vesical artery (as a common pedicle with the superior vesical artery), and 27.5% of PA originated from the pudendal artery. Unlike reported by Bilhim T et al. [23] and others [10,24], we did not found that the prostatic arteries originated from the obturator artery, inferior gluteal artery, and superior gluteal artery.

A modified embolization protocol, which developed was based on others work [13] and our early clinical experience of PAE, was used in this study. We started embolization with smaller-sized PVA particles (50-μm) for the distal embolization, and ended with larger (100-μm) for the proximal embolization. Our data showed that the mean PV was decreased from 118.0 ± 35.0 mL to 69.0 ± 18.0 mL (a mean reduction of 41.5%) after PAE at 24-month follow up. The reduction rate was higher than those of previous reports by Bagla et al. [11] with a mean reduction of 18% and by Pisco et al. [9] with a mean reduction of 20%. Using the “standard technique” and 100-300 μm particles size, the infarcts have been seen in only 70.6% of the patients with a mean infarction rate of 30%-50% after PAE [9,25]. In the present study, we have observed infarcts area ≥50% in all patients with clinical success as measured by MRI. In addition, we have observed that serum total PSA values increased significantly at 24 h after embolization, with a mean 21.9 times relative to the mean baseline values; these also suggested that greater prostate infarction occurred after PAE with the smaller size particles.

It is reasonable to assume that smaller-sized particles may induce greater ischemia with a more distal penetration into the prostate microvasculature [13], and hence lead to a better clinical outcome. Because BPH develops primarily in the peri-urethral region of the prostate, therefore embolization of this part is important for improvement of LUTS. From previous studies [9,13], we knew that 100-μm PVA particles could be used safely for PAE without untargeted embolization. Anatomically, the prostatic part of the urethra is supplied by a branch of prostatic artery, both in dogs and in humans, with a diameter of 40–60 μm [26]. Based on these data, particles with 50-μm in size may penetrate into the peri-urethral region of the prostate, with a better result than that of particles ≥100-μm in size. However, untargeted embolization and injury of the urethral wall should be concerned using the small sized particles. In the present study, no major complications were observed from PAE in any patient treated, the minor complication rates were comparable to previously reported results [9-11], and all minor complications could be addressed with conservative care, showing that PAE with the combination of 50-μm and 100-μm particles is a safe procedure.

Bilateral PAE appears to produce better results than that of unilateral PAE. According to the reported by Bilhim T et al. [27], good clinical outcomes and improvements in urodynamic data could be achieved even in patients who underwent unilateral PAE. Another series reported by the same authors [28] showed that unilateral PAE might lead to moderate clinical relief with 8% PV reduction and 18% reduction in PSA. The authors suggested that the anastomoses between prostatic arteries from both pelvic sides, presented in as many as 20% of individuals, may partially explain these results [29]. In our study, of the 8 patients with unilateral PAE, Only two patients had clinical improvement during a 24-month follow-up. Carnevale FC et al. [30] reported one patient had unilateral PAE with continuous prostate reduction until 12 months follow-up (maximum of 27.8% reduction at the 6-month follow-up) and re-growth to the initial size at the 3-year follow-up. Therefore, the bilateral PAs and any other prostatic branches should be embolized to achieve optimal prostate ischemia, resulting in volume reduction for better long-term results.

No serious complications or adverse events in the performance of PAE were observed in the present series. The incidence of minor complications (ie., transient hematuria, hemospermia, and rectal bleeding) after PAE in the patients with large BPH was similar to those of previous reports [9-12]. In comparison with others reports [9,11,21], however, the acute urinary retention (AUR) after PAE was relatively high (28.4%) in our series; this may explained by the large volume BPH nature and edema in the periurethral prostatic tissue after embolization. For management of AUR, a temporary bladder catheter and antibiotics should be maintained for 1 week after PAE under the urologist’s supervision.

There are some limitations to the present study. First, this study was a single-center experience with limited follow-up; however, continued follow-up is ongoing, and longer follow-up in our patients will bring additional information in the future. Second, the present study included only in patients with large-volume BPH and with unsuited for surgery; further analyses are necessary to establish the role of PAE in patients who are candidates for surgery, or the prostate volume less than 80 mL. Third, only PVA particle was used for our procedures; further investigation concerning different type of embolic agents are necessary. Finally, this is a non-randomised and non-comparative study. Although the results are promising more studies are needed, especially multicentre randomised controlled trials.

## Conclusions

Our clinical results shows that PAE is a safe and effective treatment method for patients with severe LUTS due to large volume BPH. PAE may play an important role in patients in whom medical therapy has failed, who are not candidates for open surgery or TURP or refuse any surgical treatment. The prostatic artery origins in the present study population were different from previously published results. Larger case series, longer follow-up time, and comparative studies with standard TURP or holmium laser enucleation of the prostate (HoLEP) are needed, not as much to evaluate safety and efficacy of PAE, but to determine which patients should undergo which treatment.

## Abbreviations

BPH: Benign prostatic hyperplasia
CB-CT: Cone-beam computed tomography
DSA: Digital subtraction angiography
HoLEP: Holmium laser enucleation of the prostate
IIEF-5: International index of erectile function short form
IPSS: International prostate symptom score
LUTS: Lower urinary tract symptoms
MRI: Magnetic resonance imaging
OP: Open prostatectomy
PAE: Prostatic arterial embolization
PAs: Prostatic arteries
PSA: Prostate specific antigen
PVA: Polyvinyl alcohol particles
PVR: Post-void residual volume
QoL: Quality of life
TURP: Transurethral resection of the prostate
